# *Ranunculus bulumei* Methanol Extract Exerts Anti-Inflammatory Activity by Targeting Src/Syk in NF-κB Signaling

**DOI:** 10.3390/biom10040546

**Published:** 2020-04-03

**Authors:** Yo Han Hong, Ji Hye Kim, Jae Youl Cho

**Affiliations:** Department of Integrative Biotechnology, Sungkyunkwan University, Suwon 16419, Korea; ghddygks12@skku.edu (Y.H.H.); kjhmlkjhml@hanmail.net (J.H.K.)

**Keywords:** *Ranunculus bulumei*, anti-inflammatory activity, NF-κB signal pathway, Src, Syk

## Abstract

(1) Background: *Ranunculus bulumei* is a flowering plant that belongs to the *Ranunculus* species. Several *Ranunculus* species, such as *R. aquatilis* and *R. muricatus*, have traditionally been used to treat fever and rheumatism throughout Asia, suggesting that plants belonging to the Ranunculus species may have anti-inflammatory effects. To our knowledge, the pharmacological activity of *R. bulumei* has not been reported. Therefore, in this study, we aim to assess the anti-inflammatory activity of a methanol extract that was derived from *R. bulumei* (Rb-ME) in macrophage-mediated inflammatory responses and to identify the molecular mechanism that underlies any anti-inflammatory action. (2) Methods: The anti-inflammatory efficacy of Rb-ME was evaluated while using in vitro and in vivo experiments. The RAW264.7 cells and peritoneal macrophages were stimulated by lipopolysaccharide (LPS). In addition, LPS-induced peritonitis and HCl/EtOH-triggered gastritis models were produced. A nitric oxide (NO) assay, real-time PCR, luciferase reporter gene assay, western blot analysis, plasmid overexpression strategy, and in vitro kinase assay were used to determine the molecular mechanisms and target molecules of Rb-ME. The phytochemical active ingredients of Rb-ME were also identified by high performance liquid chromatograph (HPLC). (3) Results: Rb-ME reduced the production of NO and mRNA expression of iNOS, COX-2, IL-1β, and IL-6 without cytotoxicity. The protein secretion of TNF-α and IL-6 was also decreased by Rb-ME. HPLC analysis indicates that quercetin, luteolin, and kaempferol are the main active ingredients in the anti-inflammatory efficacy of Rb-ME. Rb-ME also blocked MyD88-induced NF-κB promoter activity and nuclear translocation of NF-κB subunits (p65 and p50). Moreover, Rb-ME reduced the phosphorylation of IκBα, Akt, p85, Src, and Syk, which are NF-κB upstream signaling molecules in LPS-activated RAW264.7 cells. According to the in vitro kinase assay, Rb-ME directly inhibits Syk kinase activity. The oral administration of Rb-ME alleviated inflammatory responses and the levels of p-IκBα in mice with LPS-induced peritonitis and HCl/EtOH-induced gastritis. (4) Conclusions Rb-ME has anti-inflammatory capacity by suppressing NF-κB signaling and it has been found to target Src and Syk in the NF-κB pathway. Based on this efficacy, Rb-ME could be developed as an anti-inflammatory herbal medicine.

## 1. Introduction

Inflammation is a biological response that protects the body from harmful external stimuli, such as pathogens, and it aims to inhibit cell damage at an early stage, remove damaged tissue and necrotic cells in the wound, and simultaneously regenerate tissue [1]. Various leukocytes are activated by recognizing the pathogen-associated molecular patterns (PAMPs) with evolutionarily conserved structures through pattern recognition receptors (PRRs), leading to inflammatory responses. Among the PRRs, Toll-like receptors (TLRs) are the most widely studied [2,3]. Lipopolysaccharide (LPS), which is a prototypical PRR and a major component of the cell wall of gram-negative bacteria, directly activates macrophages by inducing TLR4-mediated signaling [4]. The TLR4 signal pathway involves the phosphorylation of Src, spleen tyrosine kinase (Syk), phosphoinositide-3-kinase p85 (PI3K p85), Akt, inhibitor of kappa B alpha (IκBα) and mitogen activated protein kinase (MAPK), and the activation of transcription factors, such as nuclear factor-κB (NF-κB) and activator protein 1 (AP-1). The activation of the TLR4 signal is dependent on adapter molecules, including myeloid differentiation primary response 88 (MYD88) and TIR-domain-containing adapter-inducing interferon-β (TRIF) [5,6,7]. Stimulated TLR4 signaling eventually triggers inflammation through increasing the mRNA expression of inflammatory enzymes, such as inducible nitric oxide synthase (iNOS), cyclooxygenase-2 (COX-2), and inflammatory cytokines, such as *t*umor necrosis factor-α (TNF-α), interleukin (IL)-1β, and IL-6 [8,9]. These inflammatory responses are balanced not only by the immune response, but also by the immune tolerance mechanisms that inhibit them [10]. However, poorly controlled chronic inflammation leads to a number of diseases, including cardiovascular disease, dementia, and cancer, as well as inflammatory diseases, such as gastritis and peritonitis [11].

*Ranunculus* species have been commonly used for the treatment of rheumatic, severe, and intermittent fever. In addition, *R. laetus* and *R. sceleratus* are known to have therapeutic effects on conjunctivitis in the eyes and acute icteric hepatitis, respectively [12,13,14]. Moreover, it has been reported that *R. repens* has anti-hemorrhagic effects and *R. bulbosus* is effective in the treatment of neuralgia pains and diaphoretic [15,16]. However, to our knowledge, the pharmacological efficacy of *R. bulumei* has not been reported. Therefore, this study aims to identify a safe and effective remedy for inflammatory diseases by studying the anti-inflammatory effects of methanol extracts that were derived from *R. bulumei* (Rb-ME).

## 2. Materials and Methods

### 2.1. Materials

Rb-ME was purchased from the Plant Extract Bank of the Plant Diversity Research Centre (Daejeon, Korea). A stock solution was prepared by diluting Rb-ME to 100 mg/mL while using dimethyl sulfoxide (DMSO). The RAW264.7 cells and HEK293T cells were purchased from American Type Culture Collection (ATCC) (Rockville, MD, USA). RPMI1640, DMEM, fetal bovine serum (FBS), phosphate-buffered saline (PBS), and penicillin-streptomycin solution were purchased from Hyclone (Logan, UT, USA). Lipopolysaccharide (LPS), 3-(4-5-Dimethylthiazol-2-yl)-2-5-diphenyltetrazolium bromide (MTT), N-omega-nitro-L-arginine methyl ester hydrochloride (L-NAME), DMSO, quercetin, luteolin, kaempferol, TRIzol, sodium dodecyl sulfate (SDS), and ranitidine were obtained from Sigma–Aldrich (St. Louis, MO, USA). Inhibitors for Src and Syk (pp2 and piceatannol, respectively) were purchased from Calbiochem (La Jolla, CA, USA). The cDNA synthesis kit was purchased from Thermo Fisher Scientific (Waltham, MA, USA). Forward and reverse primers for iNOS, COX-2, TNF-α, IL-1β, and IL-6 were synthesized by Bioneer (Seoul, Korea). The enzyme linked immunosorbent assay (ELISA) kits for determining the TNF-α and IL-6 protein levels were purchased from R&D systems (Minneapolis, MN, USA). The kit for the luciferase assay was purchased from Promega (Madison, WI, USA). Luciferase plasmids harboring NF-κB or AP-1 binding promoter sites were used, as reported previously [17]. A myeloperoxidase (MPO) activity colorimetric assay kit was purchased from Biovision (Milpitas, CA, USA). The polyvinylidene fluoride (PVDF) membrane was purchased from Merck Millipore (Billerica, MA, USA). Antibodies against p50, p65, Lamin A/C, Src, p-Src, Syk, p-Syk, p85, p-p85, Akt, p-Akt, IκBα, p-IκBα, and β-actin were purchased from Cell Signaling Technology (Beverly, MA, USA) and Santa Cruz Biotechnology (Santa Cruz, CA, USA).

### 2.2. Treatment of Rb-ME

Rb-ME extract was obtained in completely dried, lyophilized form after the solvent (methanol) was removed from Plant Extract Bank in the Plant Diversity Research Center (http://extract.kribb.re.kr/, Daejeon, Korea). The Rb-ME powder was dissolved in DMSO at a concentration of 100 mg/mL to make a stock solution. The experiment was performed while using DMSO (vehicle) control with the same dilution level as the negative control.

### 2.3. Cell Culture

The RAW264.7 and HEK293T cells were cultured with RPMI1640, and DMEM media, respectively, in a humidified incubator that was maintained at 5% CO_2_ and 37 °C. Cell culture media supplemented with 1% penicillin-streptomycin solution and 10% FBS (RPMI1640) or 5% FBS (DMEM) were used. Trypsin was used for the subculture of HEK293T cells, whereas the RAW264.7 cells were detached from the plate by a cell scraper.

### 2.4. Nitric Oxide (NO) Assay

The RAW264.7 cells or peritoneal macrophages were pre-treated with Rb-ME or L-NAME or Prednisolone (Pred) for 30 min., and then stimulated with LPS for 24 h. The supernatant (100 μL) obtained was mixed with 100 μL of Griess reagent, as reported previously [18,19]. The absorbance of this mixture was measured at 540 nm and the concentration of NO was calculated by comparison with a standard curve.

### 2.5. MTT Assay

RAW264.7 cells, peritoneal macrophages, and HEK293 cells were dose-dependently treated with Rb-ME for 24 h. Subsequently, 10 μL of MTT solution was added and incubated for 3 h and the reaction was stopped by 15% SDS, as reported previously [20]. The samples were then incubated for an additional 24 h. The absorbance of MTT formazan was measured at a wavelength of 540 nm.

### 2.6. Animals

The C57BL/6 mice and ICR mice were purchased from Daehan Biolink (Chungbuk, South Korea) and housed 6–8 mice per cage in a 12 h light/dark cycle. The care of animals was based on guidelines that were issued by the National Institute of Health for the Care and Use of Laboratory Animals (NIH Publication 80–23, revised in 1996). The study was conducted according to the guidelines that were established by the Institutional Animal Care and Use Committee (IACUC) at Sungkyunkwan University. The IACUC number was SKKUIACUC2018-10-16-1.

### 2.7. Isolation of Peritoneal Macrophages

Male C57BL/6 mice received a 1 mL intraperitoneal (IP) injection of sterile 4% thioglycollate broth (Difco Laboratories, Detroit, MI, USA). After four days, peritoneal macrophages were obtained via IP lavage. The isolated peritoneal macrophages (1 × 10^6^ cells/mL) were washed with RPMI 1640 medium and they were cultured for 4 h at 37 °C in 5% CO_2_ in a humidified incubator.

### 2.8. High-Performance Liquid Chromatography (HPLC) Analysis

HPLC analysis was performed, as described previously, to characterize the phytochemical properties of Rb-ME [21]. The standard compounds included quercetin, luteolin, and kaempferol. For analysis, a system equipped with a KNAUER (Wellchrom) HPLC-pump K-1001, a Wellchrom high-speed scanning spectrophotometer K-2600, and a four-channel deaerator K-500 and a Phenomenex Gemini C18 ODS (5 μm) column was used. Solvent A (0.1% H_3_PO_4_ in H_2_O) and solvent B (acetonitrile) were used as elution solvents.

### 2.9. Semi-Quantitative RT-PCR

RAW264.7 cells were pre-treated with Rb-ME for 30 min. and then incubated with LPS for 6 h. The total RNA was isolated with TRIzol reagent according to the manufacturer’s instructions. Semi-quantitative PCR was performed as reported previously [22]. Table 1 lists the sequences of primers used in this study.

### 2.10. Enzyme Linked Immunosorbent Assay (ELISA)

The RAW264.7cells were pretreated with various doses (0–100 µg/mL) of Rb-ME for 30 min. and then additionally treated with LPS (1 µg/mL) for 24 h, respectively. The protein levels of TNF-α and IL-6 released from the RAW264.7 cells in culture supernatant were determined by ELISA kit, according to the instructions from the manufacturer.

### 2.11. Preparing Whole or Nuclear Lysates

The cells were washed with cold PBS containing 1 mM sodium orthovanadate and lysed using a sonicator (Thermo Fisher Scientific, Waltham, MA, USA) in ice-cold modified RIPA buffer (50 mM Tris-HCl, pH 7.4, 1% Nonidet P-40, 0.25% sodium deoxycholate, 150 mM NaCl, 1 mM Na_3_VO_4_, and 1 mM NaF), including protease inhibitors (2 mM PMSF, 100 μg/mL leupeptin, 10 µg/mL pepstatin, 1 μg/mL aprotinin, and 2 mM EDTA) for 30 min. with rotation at 4 °C to obtain total cell lysate. Lysates were refined by centrifugation at 16,000× *g* for 10 min. at 4 °C and stored at −20 °C until use. To prepare nuclear lysate, a three-step procedure was used. First, the cells were collected and lysed with 500 mL lysis buffer (50 mM KCl, 0.5% Nonidet P-40, 25 mM HEPES, 1 mM phenylmethylsulfonyl fluoride, 10 μg/mL leupeptin, 20 μg/mL aprotinin, and 100 μM 1,4-dithiothreitol) on ice for 4 min. Second, the lysates were centrifuged at 16,000× *g* for 1 min. The pellet that was obtained from lysates was washed with washing buffer (lysis buffer without Nonidet P-40). Finally, the pellet containing nuclei was incubated with an extraction buffer (lysis buffer with 500 mM KCl and 10% glycerol). The nuclei/extraction buffer mixture was frozen at −80 °C and then centrifuged at 16,000× *g* for 5 min. The supernatant was collected as a nuclear extract.

### 2.12. Western Blotting

Cell lysates, including total or nuclear fractions, were analyzed using 7–15% SDS-polyacrylamide gel electrophoresis (PAGE) [23]. The separated proteins were transferred onto a polyvinylidene difluoride (PVDF) membrane and the PVDF membrane was blocked with BSA to reduce the nonspecific antibody reactions. The membrane was incubated overnight with primary antibody in BSA. It was washed three times with Tris-buffered saline with Tween 20 (TBST) and then probed with a secondary antibody conjugated with horseradish peroxidase in BSA for 1 h. The immunoreactive bands were detected while using an enhanced chemiluminescence kit (Pierce ECL Western blotting substrate, Thermo Scientific, Waltham, MA, USA). Two different blots were obtained from two independent Western blotting analysis. Band intensity was measured and quantified using image J.

### 2.13. Luciferase Assay

The HEK293T cells were seeded at 1 × 10^6^ cells/mL in 24-well plates and then cultured for 18 h. Subsequently, the cells were transfected with plasmids encoding a luciferase gene under an NF-κB promoter (NF-κB-Luc) or AP-1 promoter (AP-1-Luc). MyD88 or TRIF genes were co-transfected to activate the luciferase genes. Transfections were performed using the polyethylenimine (PEI) method, as reported previously [24]. The transfected cells were stabilized for 24 h and then treated with Rb-ME for another 24 h. Luciferase activity was measured with a Luciferase Assay System, as described previously [25].

### 2.14. Plasmid Transfection

HEK293T cells (1 × 10^6^ cells/mL) were transfected with HA-Src or Myc-Syk genes for 24 h using PEI to induce Src and Syk-mediated NF-κB signaling. The cells were incubated with Rb-ME for 24 h. The levels of phosphorylated p85 and IκBα, total forms of Src, Syk, p85, IκBα, HA and Myc, and *β*-actin were visualized by immunoblotting.

### 2.15. In Vitro Kinase Assay

A kinase profiler service from Millipore was used. An Rb-ME stock solution was prepared to 50 × final assay concentration in 100% DMSO to assess the direct inhibitory effect of Rb-ME against Src and Syk. The reaction was initiated by incubating with Mg/ATP mixture. After 40 min. at room temperature, the reaction was stopped by adding 0.5% phosphoric acid. Then, 10 μL of the reaction solution was spotted onto a P30 filtermat and washed 4 times for 4 min. in 0.425% phosphoric acid and once in methanol before drying and measuring by scintillation counting.

### 2.16. LPS-Induced Peritonitis In Vivo Model

A protocol using C57BL/6 mice (10 mice/group) was followed to generate a peritonitis mouse model, as reported previously [26]. Rb-ME (50 and 100 mg/kg) that was suspended in 0.5% sodium carboxymethylcellulose (CMC) was orally administered once per day for five days. Acute peritonitis was evoked by an IP injection of 1.0 μL (10 μg/kg) LPS on day 4 after Rb-ME administration. The peritoneal fluid was harvested by peritoneal lavage using sterile PBS on day 5. NO production and the number of leukocytes in peritoneal exudates were measured by NO assay and counted while using a Neubauer chamber after staining with Turk solution.

### 2.17. HCl/EtOH-Gastritis In Vivo Model and Measurement of MPO Activity

ICR mice (four mice/group) were used for generating an acute gastritis in vivo model, as previously reported [27]. Rb-ME (50 and 100 mg/kg) or ranitidine (40 mg/kg) was orally administered to ICR mice twice a day for 2 days. One hour after the final administration, acute gastritis was induced by a 300 μL oral administration of 60% ethanol in 150 mM HCl. One hour later, the mice were anesthetized with isoflurane and sacrificed. Subsequently, the redness of gastric mucosal lesions was observed.

MPO activity in stomach tissues that were obtained from gastritis mice was measured using an MPO activity colorimetric assay kit, as outlined previously [28].

### 2.18. Statistical Analysis

For MTT assay and NO assay, two independent experiments were performed and each experimental group has ten (10) parallel wells to ensure the reliability of the results. For the ELISA and luciferase assay, two independent experiments were performed and six (6) parallel wells were used in each experimental group. In PCR, each experimental group was performed in triplicate using lysates that were obtained from three independent experiments. Two independent experiments were performed, and band intensity was measured and quantified using image J for Western blot analysis. Peritonitis and gastritis in vivo experiments were performed with ten (10) or four (4) mice per groups, respectively. In this study, all of the data are presented as means ± standard deviation (SD) obtained from each experiments. Analysis of variance (ANOVA) with Scheffe’s post hoc test or the Kruskal–Wallis/Mann–Whitney tests were used to compare the data and assess the significance of group differences. A *p* value less than 0.05 indicates statistical significance.

## 3. Results

### 3.1. Inhibitory Effect of Rb-ME on NO Production in RAW264.7 Cells and Peritoneal Macrophages

**Figure 1:**
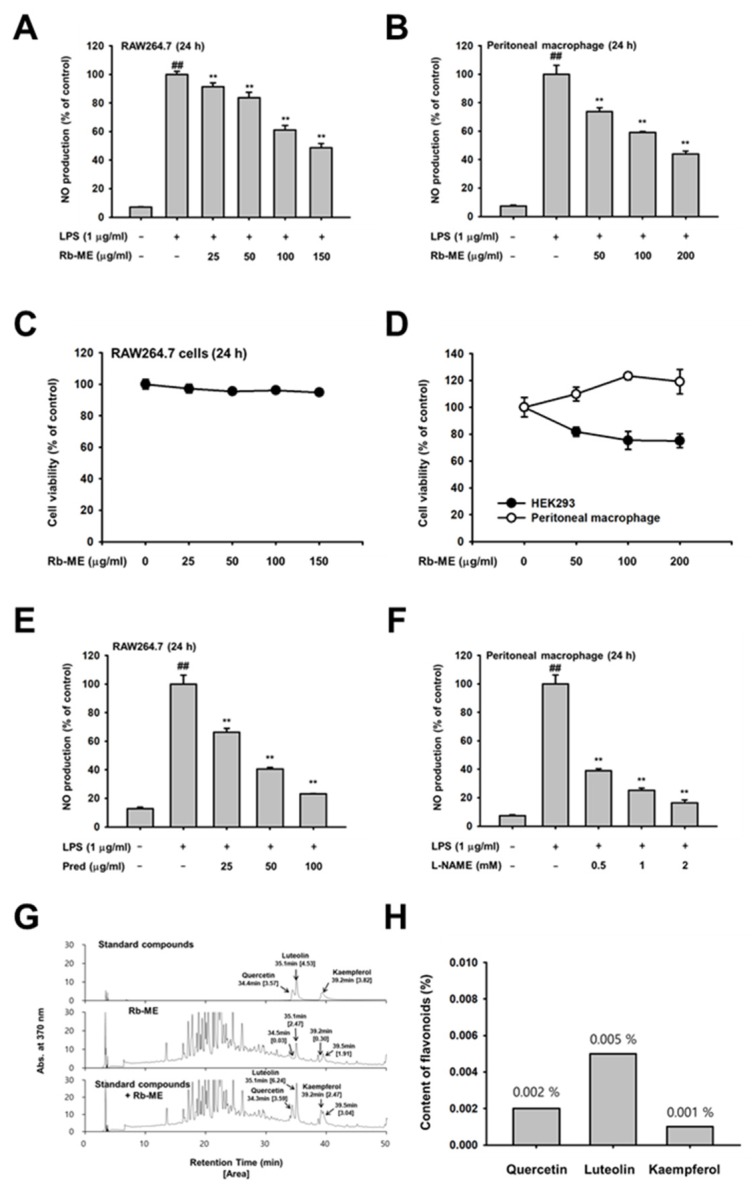
Inhibitory effect of *R. bulumei* (Rb-ME) on nitric oxide (NO) production in macrophages. (**A** and **B**) LPS-stimulated RAW264.7 cells (**A**) and peritoneal macrophages (**B**) were analyzed by NO assay in the presence or absence of Rb-ME. (**C** and **D**) Viability of Rb-ME-treated RAW264.7 cells (**C**), peritoneal macrophage, and HEK 293 cells (**D**) were measured by 3-(4-5-Dimethylthiazol-2-yl)-2-5-diphenyltetrazolium bromide (MTT) assay. (**E** and **F**) Levels of NO production in Pred-treated RAW264.7 cells (**E**) and L-NAME-treated peritoneal macrophages (**F**) in indicated doses were assessed by using a NO assay. (**G** and **H**) Active phytochemical constituents present in Rb-ME have been identified by HPLC. The profiles of Rb-ME components were analyzed by comparison with profiles obtained from standard samples of quercetin, luteolin, and kaempferol, which consisted of area and concentration. The data presented in (**A**), (**B**), (**C**), (**D**), (**E**), and (**F**) are shown as the means ± SD of two independent experiments and each experimental group was performed with ten (10) parallel wells to ensure the reliability of the results. ^##^ *p* < 0.01 compared to untreated group, and * *p* < 0.05 and ** *p* < 0.01 compared to control group treated with LPS alone.

We observed the effect of Rb-ME on the production of nitric oxide (NO), an inflammatory mediator, in LPS-treated RAW264.7 cells and peritoneal macrophages, to evaluate the anti-inflammatory efficacy of Rb-ME. NO production in Rb-ME-treated RAW264.7 cells and primary peritoneal macrophages was significantly reduced in a dose-dependent manner (Figure 1A,B). Rb-ME does not exhibit cytotoxicity at the treated concentration in the cells (Figure 1C,D). Prednisolone (Pred), a medicine that is used in various inflammatory diseases, and L-NAME, a well-known NO inhibitor, also suppressed NO production in LPS-treated RAW264.7 cells and peritoneal macrophages, respectively (Figure 1E,F). We then performed HPLC analysis using quercetin, luteolin, and kaempferol as standard compounds to determine the active phytochemical composition of Rb-ME (Figure 1G). The HPLC results showed that Rb-ME contained 0.002% quercetin, 0.005% luteolin, and 0.001% kaempferol, implying that luteolin is one of the major active phenolic components of Rb-ME (Figure 1H).

### 3.2. Suppressive Effect of Rb-ME on Inflammatory Gene Expressions at a Transcriptional Level

**Figure 2:**
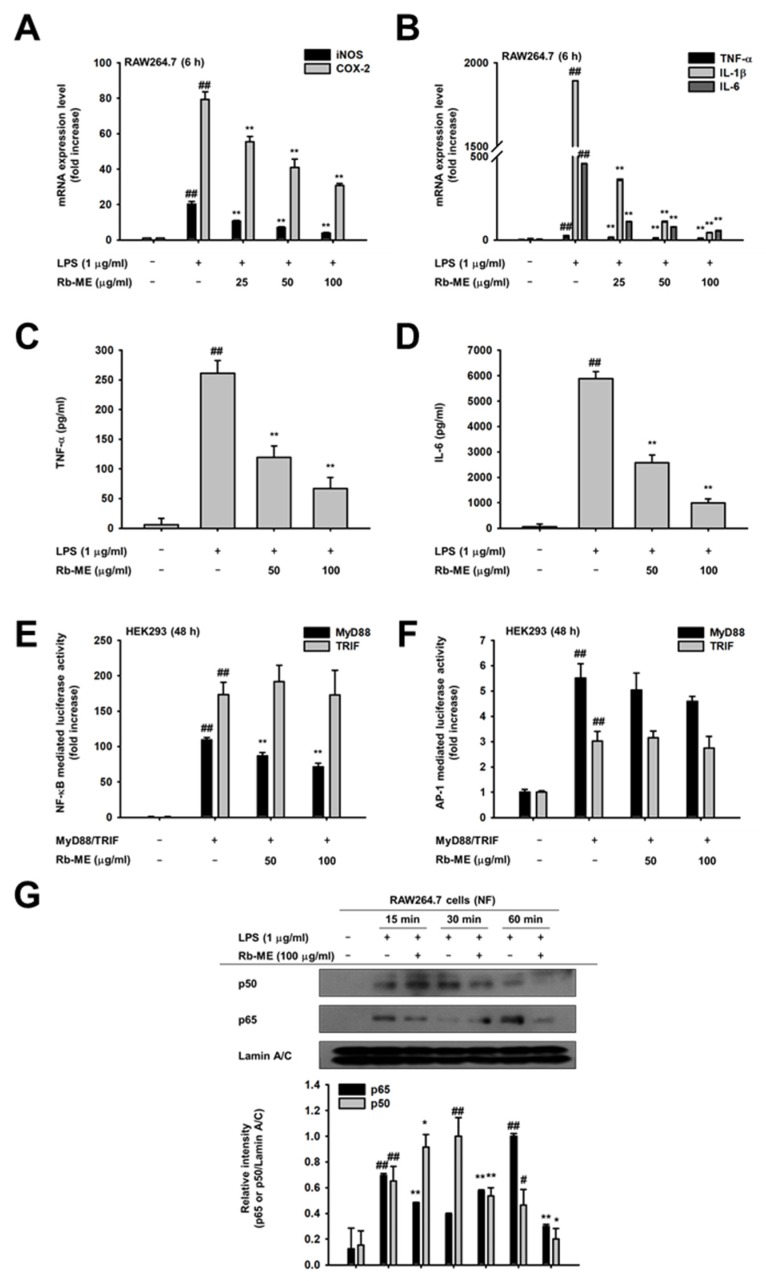
Effect of Rb-ME on the expression of inflammatory biomarkers at a transcriptional level. (**A** and **B**) RAW264.7 cells were pre-treated with Rb-ME (0–100 µg/mL) for 30 min. and then stimulated with LPS (1 µg/mL) for 6 h. The mRNA expression levels of proinflammatory cytokines, including iNOS, COX-2, TNF-α IL-1β and IL-6, were measured using semi-quantitative RT-PCR. (**C** and **D**) RAW264.7cells were pretreated with doses of Rb-ME (0-100 µg/mL) for 30 min. and then additionally treated with LPS (1 µg/mL) for 24 h, respectively. Protein levels of (**C**) TNF-α and (**D**) IL-6 released from the RAW264.7 cells were determined by ELISA kit. (**E** and **F**) HEK 293 cells were transfected with NF-κB-Luc and β-gal constructs with Flag-MyD88 or CFP-TRIF overexpressed (**E**). Flag-MyD88 or CFP-TRIF-transfected HEK 293 cells were overexpressed with AP-1-Luc and β-gal (**F**). Then, HEK 293 cells were additionally treated with Rb-ME (0–100 µg/mL) for 24 h. Luciferase activity was normalized using β-gal values after luminometer measurements. (**G**) Rb-ME (0 or 100 μg/mL) treated RAW264.7 cells were stimulated with LPS for the indicated times. Western blot analysis was performed with nuclear fractions to determine the nuclear translocation levels of NF-κB subunits (p65 and p50). Lamin A/C was used as a loading control for the nuclear fraction. The data presented at (**A**) and (**B**) are expressed as the means ± SD of three independent experiments. The data presented in (**C**), (**D**), (**E**) and (**F**) are expressed as the means ± SD of two independent experiments and each experimental group was performed with six (6) parallel wells to ensure the reliability of the results. The data presented in (**G**) is a representative of two independent experiments. # *p* < 0.05 and ## *p* < 0.01 compared to untreated group, and * *p* < 0.05 and ** *p* < 0.01 compared to control groups treated with LPS, MyD88 or TRIF alone.

Semi-quantitative RT-PCR was conducted to assess the effect of Rb-ME on inflammatory enzymes and cytokines. When LPS-stimulated RAW264.7 cells were treated with Rb-ME, the changes were observed in the mRNA levels of iNOS and COX-2. The *iNOS* and *COX-2* genes are known to be overexpressed during infection and they play an important role in the inflammatory response. As a result, it was confirmed that Rb-ME strongly reduced the expression of these genes (Figure 2A). The expression of the proinflammatory cytokines, such as IL-1β, IL-6, and TNF-α, were also significantly suppressed by Rb-ME (Figure 2B). In addition, Rb-ME diminished the secretion of TNF-α and IL-6 in a dose-dependent manner (Figure 2C,D). We evaluated whether Rb-ME can modulate these two transcription factors since Rb-ME could regulate the expression of inflammatory genes at the transcription level and NF-κB and AP-1 represent transcription factors that regulate the expression of these genes [29,30]. We overexpressed MyD88 or TRIF in HEK 293 cells and then performed a luciferase assay using plasmids harboring NF-κB or AP-1 promoter sites (NF-κB-Luc or AP-1-Luc) to mimic LPS-treated conditions in RAW264.7 cells. Luciferase activities of NF-κB-Luc and AP-1-Luc were increased by MyD88 and TRIF (Figure 2E,F). However, Rb-ME reduced the activity of NF-κB-Luc, which was increased by MyD88 (Figure 2E), suggesting that Rb-ME targets MyD88-dependent NF-κB signaling. In agreement with these results, Rb-ME blocked the translocation of p50 and p65, and subunits of NF-κB, into the nucleus of LPS-treated RAW264.7 cells (Figure 2G).

### 3.3. Effect of Rb-ME on the NF-κB Signaling Pathway

**Figure 3:**
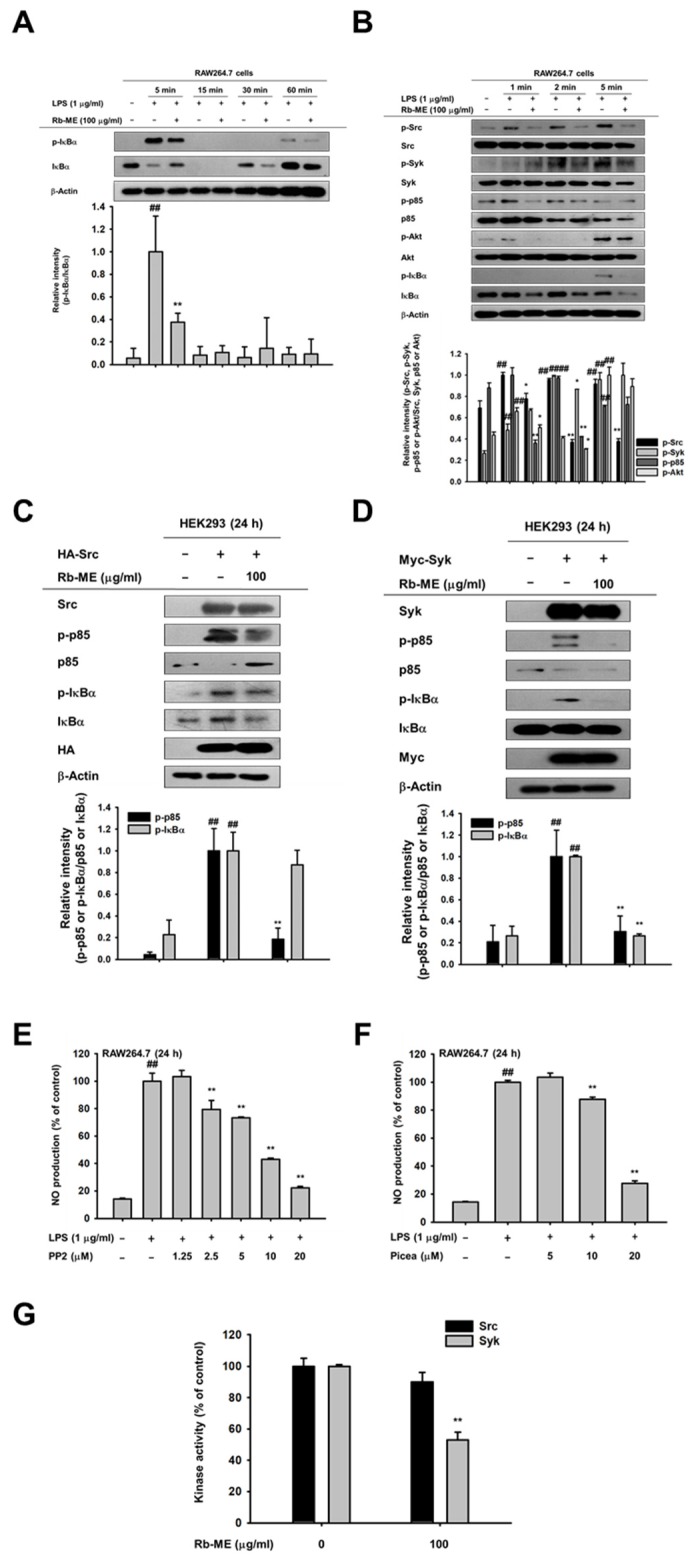
Suppressive effect of Rb-ME on the NF-κB signal pathway. (A and B) Western blotting was performed using the whole lysates obtained from LPS-treated RAW264.7 cells for the indicated time in the presence or absence of Rb-ME (100 μg/mL). The levels of phosphorylated and total forms of IκBα (**A**), and Src, Syk, p85, Akt, IκBα, and β-actin (**B**) were determined by using specific antibodies for phospho- or total-proteins. (**C** and **D**) HEK 293 cells were transfected with HA-Src (**C**) or Myc-Syk (**D**), followed by treatment with Rb-ME (100 μg/mL) for 24 h. Src, Syk, HA, Myc, and β-actin, as well as phospho- or total-forms of p85 and IκBα were assessed by immunoblotting. (**E** and **F**) NO production was analyzed by a NO assay in LPS-stimulated RAW264.7 cells after treatment with PP2, a Src inhibitor (**E**), or Picea, a Syk inhibitor (**F**). (**G**) The effects of Rb-ME on Src and Syk activity were examined using an in vitro kinase assay with purified Src and Syk. The data presented in (**A**), (**B**), (**C**), and (**D**) are a representative of two independent experiments. Relative intensity is expressed as means ± SD of data measured and quantified using image J. The data presented in (**E**) and (**F**) are expressed as the means ± SD of two independent experiments and each experimental group was performed with ten (10) parallel wells to ensure the reliability of the results. # *p* < 0.05 and ## *p* < 0.01 compared to untreated group, and * *p* < 0.05 and ** *p* < 0.01 compared to control groups treated with LPS, Src, or Syk alone.

Next, to understand the molecular mechanisms underlying the pharmacological activity of Rb-ME, we studied the effect of Rb-ME on upstream signaling molecules of NF-κB. As shown in Figure 3A, Rb-ME inhibited phosphorylation of IκBα at 5 and 30 min. in LPS-treated RAW264.7 cells. However, IκBα and p-IκBα bands were not consistently detected at 15 min. (Figure 3A) due to degradation of IκBα protein at 15 min. and rapid recovery of the protein at 30 min. after LPS stimulation, as reported previously [31]. Therefore, to determine the target molecules of Rb-ME, the phosphorylation levels of proteins which are responsible for IκBα activation in NF-κB signaling were observed at 1, 2, and 5 min. after LPS treatment. The phosphorylation of Akt, p85, Syk, and Src was decreased by Rb-ME (Figure 3B). On the basis of these results, we predicted that Src and Syk [31], which are activated the earliest in IκBα phosphorylation, could be targets of Rb-ME. To confirm this, we activated Src and Syk in HEK 293 cells through the transfection of Src and Syk plasmids and observed that Rb-ME strongly blocked the phosphorylation of p85 and IκBα, which were induced by Src and Syk overexpression (Figure 3C,D). In addition, the Src and Syk inhibitors PP2 and piceatannol suppressed LPS-induced NO production (Figure 3E,F). Subsequently, we analyzed the direct effect of Rb-ME on Src and Syk by using an in vitro kinase assay. Interestingly, Rb-ME only blocked the activity of Syk kinase (Figure 3G), which indicated that Rb-ME targets both Src and Syk, but especially Syk. Rb-ME inhibits Syk kinase activity by direct binding.

### 3.4. Anti-Inflammatory Effect of Rb-ME in LPS-Induced Peritonitis and HCl/EtOH-Induced Gastritis In Vivo Models

**Figure 4:**
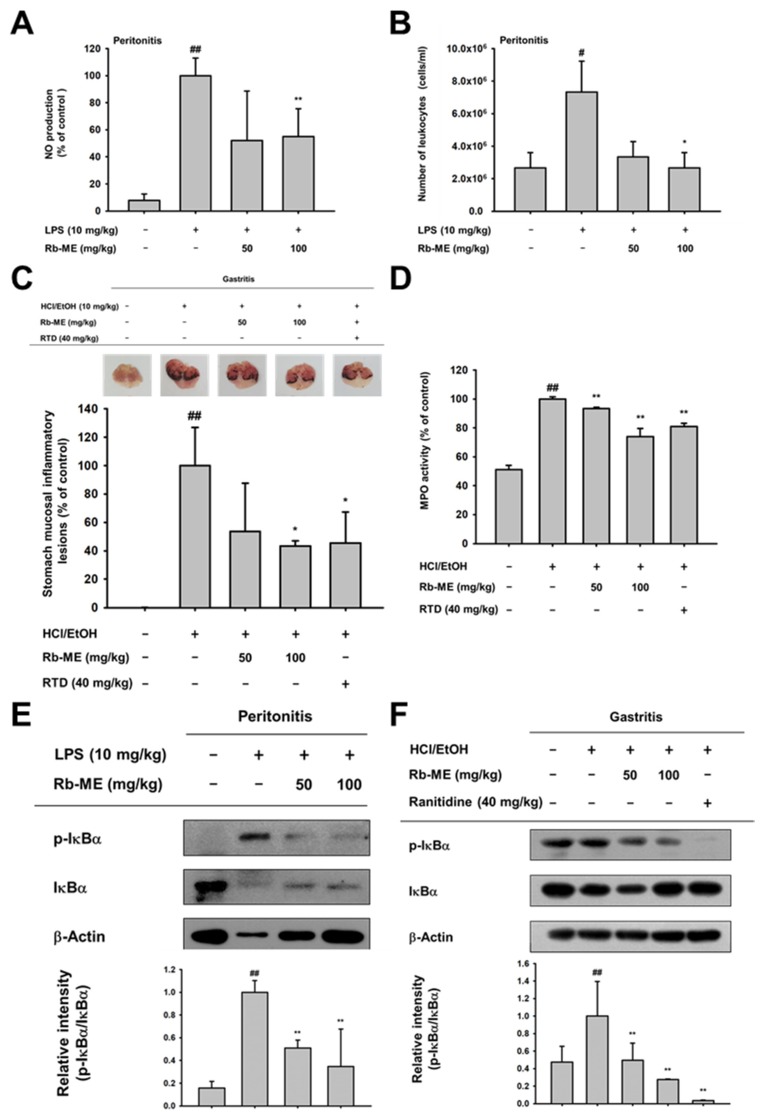
Anti-inflammatory effect of Rb-ME in peritonitis and gastritis models in vivo. (**A** and **B**) Rb-ME (50 and 100 mg/kg) was orally administered to C57BL/6 mice once a day for 5 days, and peritonitis was induced by intraperitoneal injection of LPS (10 μg/kg) for 1 day before mice were sacrificed. The effect of Rb-ME on NO production was analyzed in peritoneal exudates by using a NO assay (**A**). The number of leukocytes in peritoneal exudates was counted by using and a Neubauer chamber after staining with Turk solution (**B**). (**C** and **D**) Rb-ME (50 and 100 mg/kg) or ranitidine (40 mg/kg) was orally administered to ICR mice two times a day for two days. Gastritis was induced by HCl/EtOH injection 1 h before sacrifice. Gastric inflammatory lesions were taken with a digital camera and then quantitatively measured using Image j (**C**). Myeloperoxidase (MPO) activity was determined in total lysates obtained from the stomach tissue of gastritis mice treated with Rb-ME or ranitidine using an MPO activity colorimetric assay kit (**D**). (**E** and **F**) Immunoblotting assay was performed with whole lysates obtained from peritoneal exudates of peritonitis mice (**D**) or stomach tissue of gastritis mice (**E**). Phospho- or total-forms of IκBα and β-actin were detected using specific antibodies. The data presented in (**A**), (**B**), (**C**), and (**D**) are expressed as the mean ± SD of experiments that were performed with ten (**A** and **B**) and four (**C** and **D**) mice per group. The data presented in (**E**) and (**F**) are a representative of two independent experiments. Relative intensity is expressed as means ± SD of data measured and quantified using image J. # *p* < 0.05 and ## *p* < 0.01 as compared to untreated group, and * *p* < 0.05 and ** *p* < 0.01 compared to control groups treated with LPS or HCl/EtOH alone.

In vivo models of peritonitis and gastritis were created to evaluate whether Rb-ME is indeed pharmaceutically effective in inflammatory diseases. The peritonitis mouse model was induced by the IP administration of LPS. Oral administration of Rb-ME (100 mg/kg) significantly reduced the production of NO in peritoneal macrophages obtained from the peritonitis model (Figure 4A). The number of leukocytes in peritoneal exudates was also diminished in the Rb-ME-administrated group (Figure 4B). In addition, Rb-ME exerts an inhibitory effect on the damage that occurs in the HCl/EtOH-induced gastritis model. Bleeding and redness occurred in the gastric mucosa of the control group because of the damage induced by HCl/EtOH, according to visual observations. On the other hand, hemorrhagic mucosal damage was reduced in the positive control (ranitidine) and Rb-ME treated groups (Figure 4C). Rb-ME also decreased the gastric mucosal MPO activity (Figure 4D). Finally, we evaluated whether Rb-ME targets NF-κB signaling in experimental models in vivo. Similar to the results in RAW264.7 cells (Figure 3A,B), Rb-ME exerts an inhibitory effect on the phosphorylation of IκBα in peritonitis model-derived peritoneal macrophages and gastric tissue of the gastritis model (Figure 4E,F). These results imply that Rb-ME alleviates inflammatory responses through the regulation of NF-κB signaling.

## 4. Discussions

*Ranunculus* species have been commonly used in traditional medicines to treat rheumatism, intermittent fever, and rubefacient [32]. However, in some plants, like *R. arvensis*, *R. bulbosus, R. ficaria, R. sardous,* and *R. sceleratus,* caution must be required when using for medicinal and edible purposes, since crushing leaves produce protoanemonin, a toxic substance, which damages the skin and mucous membranes [33,34]. In this study, Rb-ME, which is a methanol extract of *Ranunculus bulumei* whose pharmacological activity has not been reported, did not show toxicity in macrophages, such as RAW264.7 cells and peritoneal macrophages and in mice (data not shown). Therefore, we evaluated the anti-inflammatory effects of Rb-ME in macrophage-like cells, LPS-induced peritonitis mice, and HCl/EtOH-triggered gastritis mice.

However, to our knowledge, the pharmacological activity of *Ranunculus bulumei* has not been reported. Therefore, in this study, we evaluated the anti-inflammatory effects of Rb-ME in systems in vitro and in vivo using macrophage, LPS-induced peritonitis mice, and HCl/EtOH-triggered gastritis mice.

Inflammation is a part of the normal immune response, but it should be properly controlled, because chronic inflammation can lead to multiple diseases, including cancer and cardiovascular disorders. NO is one of the most notable effector molecules in the inflammatory response [35]. In the immune system, NO was initially defined as a small molecule mediator with antibacterial function that is produced by cytokine-activated macrophages [36,37]. However, the definition of NO has been extended to incorporate immune cells other than macrophages that can produce NO and it might be involved in various physiological phenomena, including immune defense, blood pressure regulation, platelet aggregation, and the regulation of cell signaling [38]. NO is produced by three types of NO synthase (NOS): endothelial (eNOS), neuronal (nNOS), and inducible (iNOS) [39]. In the immune response, large amounts of NO are mainly produced by iNOS in response to cytokine stimulation [40]. In chronic inflammation, iNOS is thought to be involved in the formation of granulomatous lesions [41]. In addition, it has been reported that L-NAME dose-dependently reduces the size of chronic inflammatory lesions in a chronic inflammation model while using rats [42]. These studies indicate that NO is associated with destructive chronic inflammation and the inhibition of NO production could have potential in treating chronic inflammatory diseases [43]. Indeed, it has been reported that some nonsteroidal anti-inflammatory drugs (NSAIDs) restrain the inflammatory response by blocking the expression of NOS [44,45,46]. However, since these drugs have side effects that are associated with the gastrointestinal tract and cardiovascular system when taken for a long time [47,48], research on the development of safe ethnopharmacological drugs are actively in progress. Interestingly, Rb-ME inhibited NO production without cytotoxicity in both LPS-stimulated peritoneal macrophage and RAW264.7 cells (Figure 1A–D). Pred, which is an inflammatory drug, and L-NAME, a NO inhibitor, also showed inhibitory effects against NO production in LPS-treated RAW264.7 and peritoneal macrophage, respectively (Figure 1E,F). In addition, Rb-ME dramatically reduced the expression of iNOS (Figure 2A). Rb-ME also suppressed the expression of COX-2, which is a key enzyme in inflammatory responses, and TNF-α, IL-1β, and IL-6, which are representative inflammatory cytokines (Figure 2B). The secretion of TNF-α and IL-6 was also dramatically diminished, which indicated that Rb-ME has a functional impact on macrophages-mediated inflammation. We used LPS-triggered peritonitis and HCl/EtOH-induced gastritis models to evaluate the in vivo anti-inflammatory effects of Rb-ME. After LPS-induced peritonitis mice received the oral administration of Rb-ME, NO production, and the number of leukocytes were reduced (Figure 4A). Furthermore, gastric mucosal damage and MPO activity were significantly diminished in the Rb-ME-administrated group of the gastritis model (Figure 4D). In particular, Rb-ME alleviated gastritis as strongly as ranitidine, which is mainly used to treat gastric ulcers or gastroesophageal reflux disease by acting as an H2 receptor blocker and inhibiting gastric acid production. These results imply that Rb-ME has anti-inflammatory efficacy.

Next, we explored the mechanisms by which Rb-ME exerts anti-inflammatory activity. The transcription factors that are involved in the expression of inflammatory genes include NF-κB and AP-1 [49,50]. Therefore, the effect of Rb-ME on NF-κB and AP-1 promoter activities was evaluated by performing a luciferase assay. The activities of NF-κB-Luc and AP-1-Luc were stimulated by MyD88 or TRIF overexpression. Intriguingly, Rb-ME was only involved in the regulation of MyD88-dependent NF-κB luciferase activity (Figure 2E,F). Rb-ME also reduced the nuclear translocation of p65 and p50, which are NF-κB subunits (Figure 2G), and phosphorylation of IκBα, Akt, p85, Syk, and Src in LPS-treated RAW264.7 cells (Figure 3A,B). In addition, the phosphorylation of IκBα was inhibited by Rb-ME in peritoneal macrophages that were derived from LPS-induced peritonitis mice and in HCl/EtOH-triggered gastritis mice (Figure 4E,F), which suggested that Rb-ME exhibits an anti-inflammatory effect by participating in the regulation of NF-κB signaling. 

It has been reported that Src and Syk are initially activated in NF-κB signaling when LPS stimulates TLR4 [51,52]. In addition, according to our results, pp2, a Src inhibitor, and piceatannol, a Syk inhibitor, can block NO production (Figure 3E,F). Furthermore, Rb-ME blocked both Src and Syk phosphorylations (Figure 3B). Based on these reports and our findings, we assumed that Src and Syk are the targets of Rb-ME. Therefore, the inhibitory effect of Rb-ME on Src and Syk-mediated NF-κB signaling was investigated. As we expected, Rb-ME clearly inhibited the phosphorylation of IκBα and p85, which are activated by Src and Syk overexpression (Figure 3C,D). However, interestingly, in vitro kinase assays showed different results for Src and Syk, indicating that Rb-ME directly suppressed Syk kinase activity, but indirectly inhibited Src kinase (Figure 3G). This result also suggests that Rb-ME targets phosphatases or kinases that modulate Src kinase instead of directly regulating Src kinase.

The inhibition of NF-κB might be an attractive anti-inflammatory strategy, given the evidence showing significant involvement of NF-κB in the development of inflammatory diseases [53,54]. However, NF-κB is a multifunctional molecule that contributes to maintaining homeostasis through the control of normal cellular responses, including cell cycle and cell death [55,56]. Thus, a balance between therapeutic effects and maintenance of normal biological responses is the most important consideration in developing NF-κB inhibition as a therapeutic agent [57]. In this regard, Rb-ME might be more advantageous in terms of safety than drugs that directly block NF-κB, because Rb-ME selectively suppresses inducible NF-κB under inflammatory conditions by inhibiting Src and Syk, upstream molecules of NF-κB signaling. 

**Figure 5:**
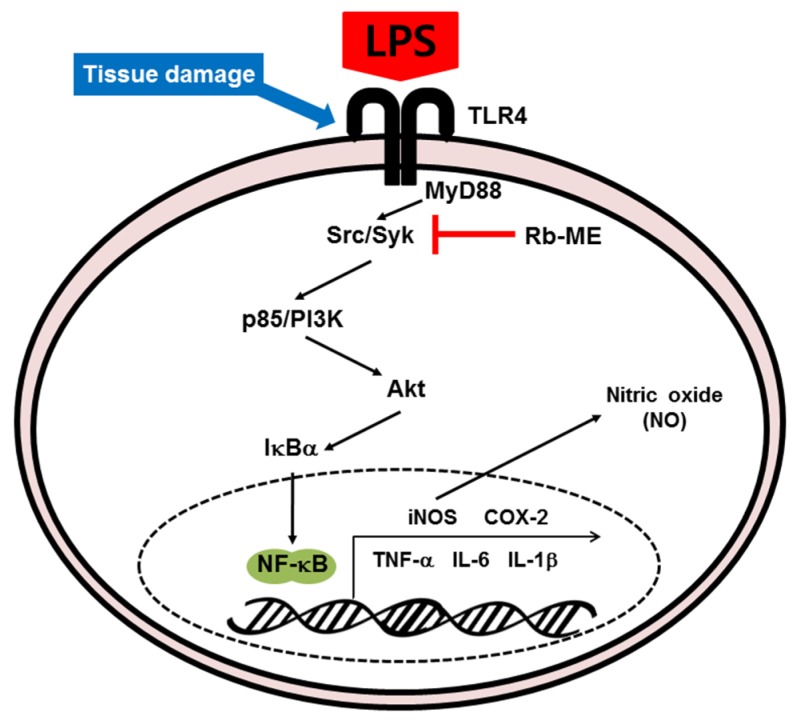
Schematic diagram of the anti-inflammatory effect of Rb-ME through inhibition of NF-κB signaling.

Taken together, Rb-ME impairs the NF-κB signal pathway, which consists of p85, Akt, IκBα, and p65/p50, via blocking Src and Syk activity. Consequently, Rb-ME reduces the expression of various inflammatory molecules, such as iNOS, COX-2, IL-1β, and IL-6, and the production of NO, which is an inflammatory mediator (Figure 5). Our results demonstrate that Rb-ME is a promising substance with efficacy as an anti-inflammatory drug.

## Figures and Tables

**Table 1:** Sequences of PCR primers used in this study.

Targets	Direction	Sequences (5′ to 3′)
iNOS	Forward	GGAGCCTTTAGACCTCAACAGA
	Reverse	TGAACGAGGAGGGTGGTG
COX-2	Forward	CACTACATCCTGACCCACTT
	Reverse	ATGCTCCTGCTTGAGTATGT
TNF-α	Forward	GCCTCTTCTCATTCCTGCTTG
	Reverse	CTGATGAGAGGGAGGCCATT
IL-1β	Forward	CAACCAACAAGTGATATTCTCCATG
	Reverse	GATCCACACACTCCAGCTGCA
IL-6	Forward	CTAGGTTTGCCGAGTAGATCTC
	Reverse	GACAAAGCCAGAGTCCTTCAGAGA
GAPDH	Forward	CAATGAATACGGCTACAGCAAC
	Reverse	AGGGAGATGCTCAGTGTTGG

## Abbreviations

PAMPs	Pathogen-associated molecular patterns
PRRs	Pattern recognition receptors
TLRs	Toll-like receptors
LPS	Lipopolysaccharide
Syk	Spleen tyrosine kinase
PI3K p85	Phosphoinositide-3-kinase p85
IκBα	Inhibitor of kappa B alpha
MAPK	Mitogen activated protein kinase
MYD88	Myeloid differentiation primary response 88
TRIF	TIR-domain-containing adapter-inducing interferon-β
COX-2	Cyclooxygenase-2
IL	Interleukin
RH	Right hand (Right side)
FRT	Front
ACC	Accessory
NO	Nitric oxide
Pred	Prednisolone
TNF-α	Tumor necrosis factor-alpha
IL	Interleukin
AP-1	Activator protein 1
IP	Intraperitoneal
NOS	NO synthases
NSAIDs	Nonsteroidal anti-inflammatory drugs
